# Radiosensitization of HER2-positive esophageal cancer cells by pyrotinib

**DOI:** 10.1042/BSR20194167

**Published:** 2020-02-18

**Authors:** Xiangyao Lian, Cuimin Zhu, Haishan Lin, Zhengxing Gao, Guangxin Li, Ninggang Zhang, Bangwei Cao, Yan Kang

**Affiliations:** 1 Cancer Center, Beijing Friendship Hospital, Capital Medical University, Beijing 100050, China; 2 Department of Oncology, Affiliated Hospital of Chengde Medical College, Chengde, Hebei 067000, China; 3 Department of Surgery, Children’s National Medical Center, WA 20010, U.S.A.

**Keywords:** esophageal cancer, HER2, pyrotinib, radiation

## Abstract

Radiation therapy is a widely used treatment for esophageal cancer. However, radiation resistance might result in a poor prognosis. Overexpression of HER2 has been related to adaptive radiation resistance. Pyrotinib is a HER2 inhibitor that shows an anti-tumor effect in breast cancer. The present study aims to explore the influence of pyrotinib combined with radiotherapy on HER2-positive esophageal cancer cells and explore the underlying mechanism. We screened two cell lines (TE-1 and KYSE30) that highly express HER2 from several human esophageal cancer cell lines. Cells were treated with pyrotinib or/and radiation. Cell proliferation, cell cycle distribution, and cell migration were measured. The protein levels involved in cell cycle and DNA repair were measured by Western blot. Results showed that pyrotinib inhibited HER2 activation and exerted an anti-proliferative effect in TE-1 and KYSE30 cells. Furthermore, it enhanced the anti-proliferative effect of radiation in these two cell lines. These effects might be via inhibiting HER2 phosphorylation, inducing G0/G1 arrest, and reducing EMT and DNA repair. Our results indicated that pyrotinib sensitivitied HER2 positive esophageal cancer cells to radiation treatment through various mechanisms. These findings may provide a new therapeutic strategy for treating HER2 positive esophageal cancer.

## Introduction

Esophageal cancer is a common malignant tumor in the digestive tract that arises in the esophagus, with a dramatic increase in new cases occurring worldwide in the recent decades. Although multiple therapeutic strategies combined with surgery, radiotherapy, and chemotherapy have been developed, the 5-year survival rate of esophageal cancer remains is still low, approximately only 15–25% [1]. Radiation therapy is a widely used treatment for esophageal cancer. However, radiation resistance might lead to radiation therapy failure and result in a unsatisfactory prognosis [2]. This may depend on the regulation of key molecules or pathways in esophageal cancer cells that are related to the cell growth, metastasis, DNA damage, and repair [3]. Thus, effective radiation sensitizers targeting these molecules and pathways might improve radiation therapy and lead to better prognosis of esophageal cancer.

The human epidermal growth factor receptor 2 (HER2) belongs to the epidermal growth factor receptor (EGFR) family, which regulates cell proliferation, migration, and differentiation. It is well documented that HER2 is involved in the pathogenesis of several human cancers, including breast cancer, gastric cancer, esophageal cancer, ovarian cancer etc. [4]. The incidence of HER2 positive in esophageal cancer ranges from 9 to 64% [5]. Several HER2-targeting therapies were developed and were shown to improve the clinical outcome in the HER2 overexpressing breast and gastric/gastroesophageal cancers [4]. Moreover, overexpression of HER2 may be also related with adaptive radiation resistance. Patients with HER2-positive breast cancer have a higher recurrence rate after combined surgery and radiation treatment [6]. *In vitro* studies found that radioresistance of breast cancer cells could be reduced by HER2 inhibition mediated by herceptin or RNA interference [7].

Pyrotinib is an irreversible HER2 inhibitor that showed anti-tumor effect on breast xenograft models that overexpress HER2 [8]. Based on promising outcome in a phase II trial, the drug was recently approved in China for conditional use combined capecitabine for the treatment of advanced or metastatic HER2-positivebreast cancer [9]. Moreover, it is currently in phase I study for treatment with HER positive gastric cancer in China and in U.S.A. [10]. However, whether this drug could also exert anti-tumor effect in esophageal cancer remains unclear. Thus, the present study tested the effect of pyrotinib combined with radiotherapy on HER2-positive esophageal cancer cells, and explored the underlying mechanism.

## Materials and methods

### Cells and treatment

Human esophageal cancer cell lines TE-1, TE-10, KYSE30, EC109, KYSE150, and KYSE450 (ATCC, Manassas, VA, U.S.A.) were cultured in DMEM complete medium containing 10%FBS (Invitrogen, U.S.A.) in 5% CO_2_ at 37°C. For Pyrotinib treatment, cells were washed with PBS and incubated with FBS-free medium containing Pyrotinib (Henrui Medicine, China) at indicated dose for 24 h. For X-ray radiation, cells were subjected to irradiation at a dose of 200 cGy/min by a 6 MV linear accelerator (Elekta, Sweded). If cells were subjected to both pyrotinib and irradiation, cells were treated with pyrotinib for 24 h (3 μg/ml for TE-1, 4 μg/ml for KYSE30 cells) followed by irradiation at indicated dose. The dosage for pyrotinib was determined by cell viability assay that showed that pyrotinib produced a cytotoxic effect on TE-1 cells with IC50 = 3.32 (Supplementary Figure S1A,B) and on KYSE30 cells with IC50 = 4.294 (Supplementary Figure S1C,D).

### Western blot

After treatment, total protein was extracted from cells using RIPA lysis buffer containing 0.2 mM PMSF. Protein samples (40 μg) were subjected to 10% SDS-PAGE and electro-transferred to PVDF membranes. The membranes were blocked by 5% skimmed milk for 2 h and then incubated with following primary antibodies at 4°C overnight: anti-EGFR (1:1000, Cell Signaling), anti-HER2 (1:1000, Cell Signaling), anti-phospho-HER2 (1:1000, Cell Signaling), anti-cyclin D1 (1:500, Abcam), anti-CDK4 (1:800, Abcam),anti-AKT (1:600, Cell Signaling), anti-pAKT (1:600, Cell Signaling), anti γ-H2AX (1:200, Cell Signaling), or anti-GAPDH (as internal control, 1:800, Abcam). After washing with TBS-T and 2-h incubation with HRP-conjugated secondary antibody, membranes were incubated with ECL reagents (Thermo Scientific), scanned and analyzed using ImageJ software.

### Colony formation assay

Cells were incubated with different doses of pyrotinib for 24 h or/and treated with X-ray irradiation with different doses and then seeded in six-well plates at 1200 cells/well. After 2 weeks, cells were fixed using methanol and stained with 0.2% Crystal Violet. The number of colonies that contain more than 50 cells was counted.

### Analysis of cell cycle distribution

Cells were subjected to pyrotinib treatment for 24 h followed by 6-Gy irradiation. After 24 h, cells were detached using 0.25% trypsin and resuspended in PBS. Cells (106 for each sample) were fixed with 70% ethanol at 4°C overnight. After washing, cells were resuspended in staining solution for 30 min at room temperature in a box avoiding light, and subjected to flow cytometer analysis (BD Biosciences, U.S.A.).

### Immunofluorescence

After treatment, cells were fixed 4% PFA and treated with 0.5% NP-40 to permeabilize for 20 min. Cells were then blocked with 1% BSA for 1 h and incubated with primary antibodies against p-AKT and γ-H2AX at 4°C overnight. After washing, cells were incubated with Alexa Fluor 568 or 488 conjugated secondary antibody for 2 h and then dyed with DAPI. Slides were examined using a Leica confocal laser scanning microscope.

### Data analysis

Data from colony formation test and cell cycle analysis were presented as mean ± SD. Student’s *t*-test was used to compare the difference between drug-treated groups and control, or between groups with single treatment and combined treatment. *P* < 0.05 was considered as significant.

## Results

### Expression of EGFR and HER2 proteins in human esophageal cancer cells

**Figure 1:**
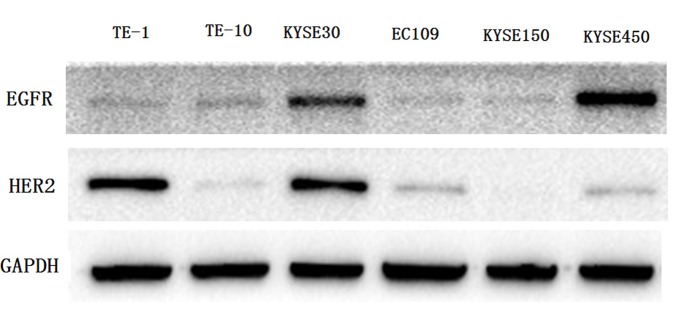
Expression of EGFR and HER2 proteins in human esophageal cancer cell lines

As shown in Figure 1, all the six human esophageal cancer cell lines presented EGFR expression. The TE-1, TE-10, KYSE30, EC109, and KYSE450 cells express different levels of HER2, during which the TE-1 and KYSE30 cells show high level HER2 expression. Therefore, these two cell lines were studied in the following experiments.

### Effect of pyrotinib on HER2 expression in human esophageal cancer cells

**Figure 2:**
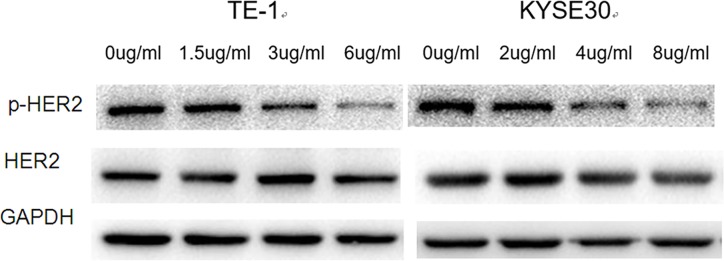
Effect of pyrotinib on HER2 expression in human esophageal cancer cell lines

In both TE-1 and KYSE30 cells, pyrotinib treatment reduced the protein levels of phosphorylated HER2 in a dose-dependent manner. However, pyrotinib did not influence the protein levels of total HER2 in these cells (Figure 2).

### Pyrotinib reduces colony formation of human esophageal cancer cells

**Figure 3:**
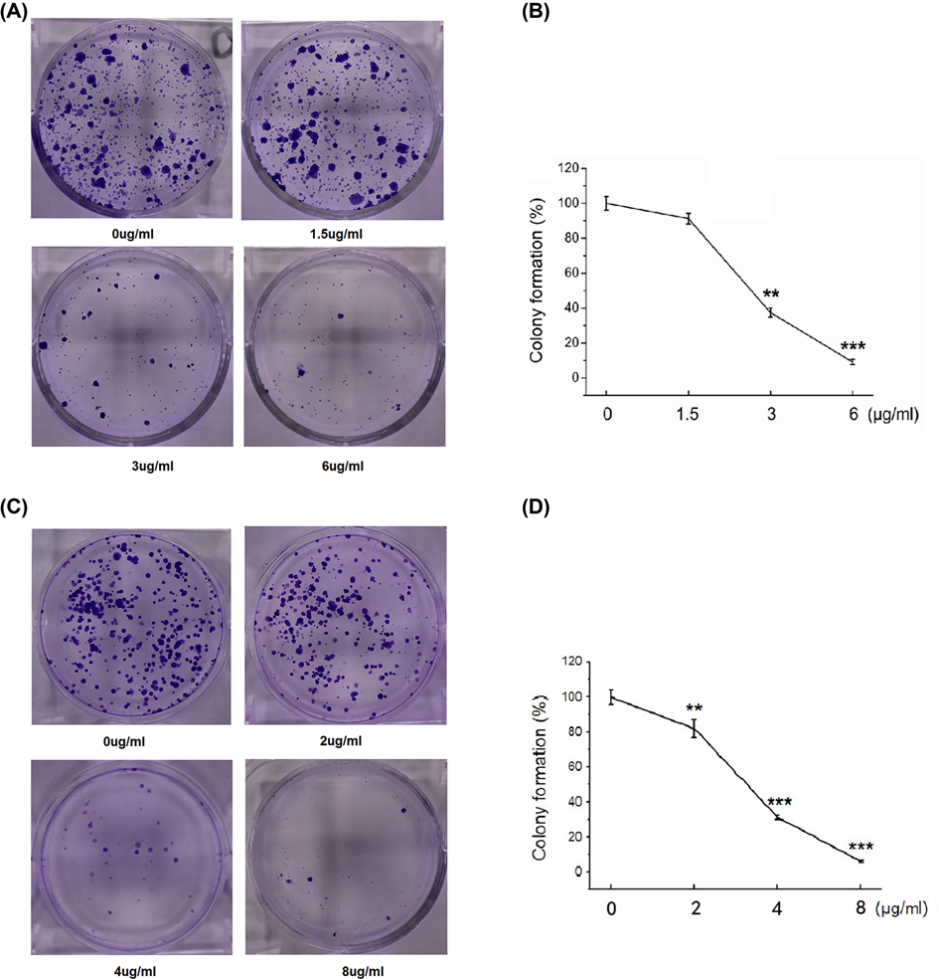
Pyrotinib reduces colony formation of human esophageal cancer cells Cells were treated with pyrotinib for 24 h. (**A**) Representative picture of colony formation assay in TE-1 cells. (**B**) Percent of colony formation of pyrotinib treated TE-1 cells. (**C**) Representative picture of colony formation assay in KYSE30 cells. (**D**) Percent of colony formation of pyrotinib treated KYSE30 cells. **P < 0.01; ***P < 0.001 compared with control group.

As shown in Figure 3, pyrotinib inhibited the colony formation of TE-1 and KYSE30 cells, in dose-dependent manners. In TE-1 cells, pyrotinib at higher doses significantly reduced colony formation, resulting in ∼40% colony formation at 3 μg/ml and ∼10% colony formation at 6 μg/ml, compared with control group (Figure 3A, B). Pyrotinib inhibited colony formation of KYSE30 cells at all doses, resulting in ∼80 colony formation at 2 μg/ml, ∼30% colony formation at 4 μg/ml and <10% colony formation at 8 μg/ml, compared with control group (Figure 3C, D).

### Pyrotinib enhances the effect of irradiation on colony formation

**Figure 4:**
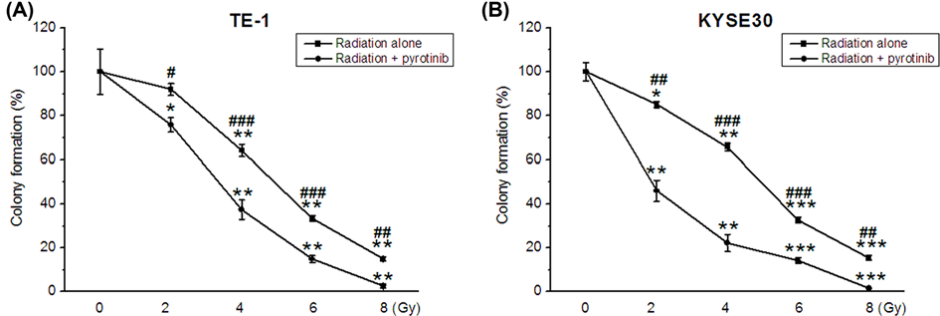
Pyrotinib enhanced the inhibitory effect of radiation on colony formation of human esophageal cancer cells Cells were treated with different doses of radiation with/without 24-h pyrotinib treatment. (**A**) Percent of colony formation of radiation and pyrotinib treated TE-1 cells. (**B**) Percent of colony formation of radiation and pyrotinib treated KYSE30 cells. *P < 0.05; **P < 0.01; ***P < 0.001 compared with non-treatment group. #*P* < 0.05; ##*P* < 0.01; ###*P* < 0.001 compared with radiation + pyrotinib group.

Radiation significantly inhibited the colony formation of TE-1 and KYSE30 cells in dose-dependent manners (Figure 4A,B). Radiation exerted inhibitory effect on colony formation in TE-1 cells with a minimal dose of 4 Gy (Figure 4A) and in KYSE30 cells the minimal dose was 2 Gy (Figure 4B). Compared with radiation treatment alone, cells treated with radiation combined with pyrotinib showed significantly reduced colony formation, indicating that pyrotinib enhanced the inhibitory effect of radiation on colony formation in these two human esophageal cancer cell lines.

### Effect of pyrotinib and irradiation on periodic distribution of human esophageal cancer cells

**Figure 5:**
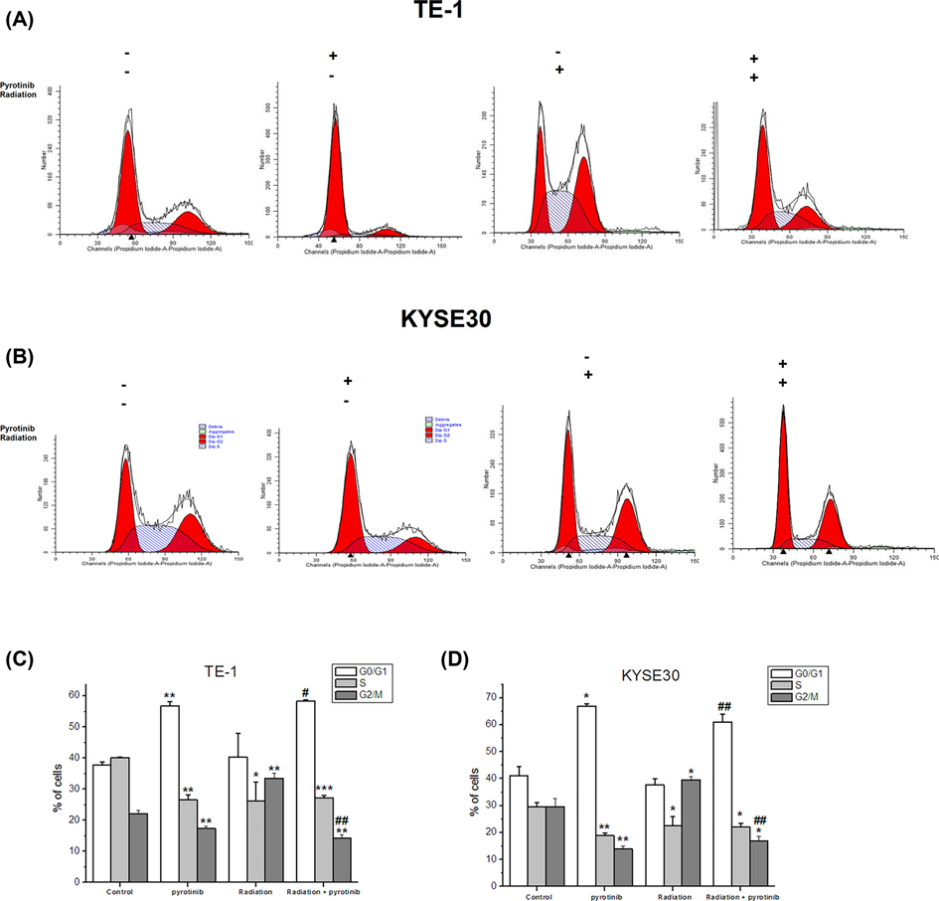
Effect of pyrotinib and irradiation on periodic distribution of human esophageal cancer cells Cells were treated with pyrotinib for 24 h or/and 6-Gy irradiation. After 24 h, cells were harvested and subjected to flow cytometry. (**A**) Flow cytometry results of cell cycle analysis of TE-1 cells. (**B**) Flow cytometry results of cell cycle analysis of KYSE30 cells. (**C**) Percent of cells in different cycle phase. *P<0.05; **P<0.01; ***P<0.001 compared with control group. #*P*<0.05; ##*P* <0.01 compared with radiation group.

Results from flow cytometry showed that irradiation significantly increased the percentage of G2/M cells in both TE-1 (Figure 5A,C) and KYSE30 cells (Figure 5B,D) compared with the control group, showing G2/M phase arrest by irradiation. Treatment with pyrotinib significantly decreased the percentage of G2/M cells while increased the percentage of G0/G1 cells compared with Radiation group (Figure 5A–D). Meanwhile, pyrotinib could also cause G0/G1 arrest and reduce G2/M phase in the cells without irradiation (Figure 5A–D).

### Effect of pyrotinib on cyclin D1 and CDK4 expression in TE-1 and KYSE30 cells

**Figure 6:**
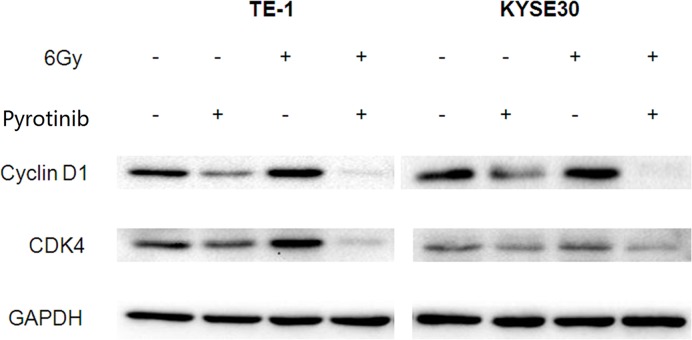
Effect of pyrotinib on expression of cyclin D1 and CDK4 in human esophageal cancer cells Cells were treated with pyrotinib for 24 h or/and 6-Gy irradiation: TE-1 (left) and KYSE30 (right).

The expression of two important cell cycle-regulating proteins, cyclin D1 and CDK4 was decreased following treatment with pyrotinib (Figure 6). Pyrotinib could also decrease the cyclin D1 and CDK4 expression in irradiation treated TE-1 and KYSE30 cells (Figure 6).

### Effect of pyrotinib on expression of DNA repair pathway proteins in human esophageal cancer cells

**Figure 7:**
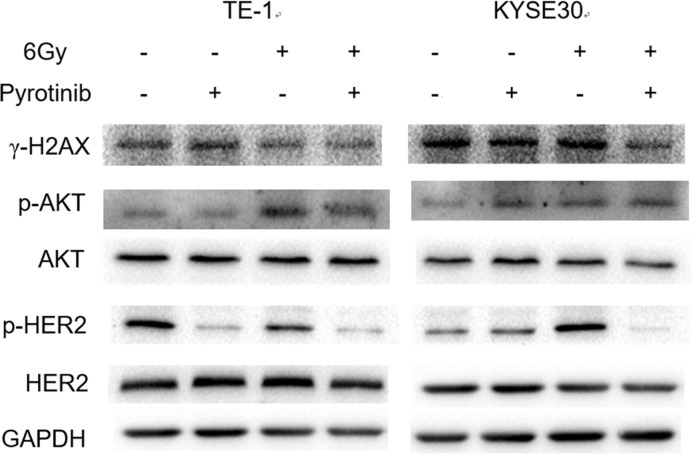
Effect of pyrotinib on expression of DNA repair pathway proteins in human esophageal cancer cells Cells were treated with pyrotinib for 24 h or/and 6-Gy irradiation: TE-1 (left) and KYSE30 (right).

**Figure 8:**
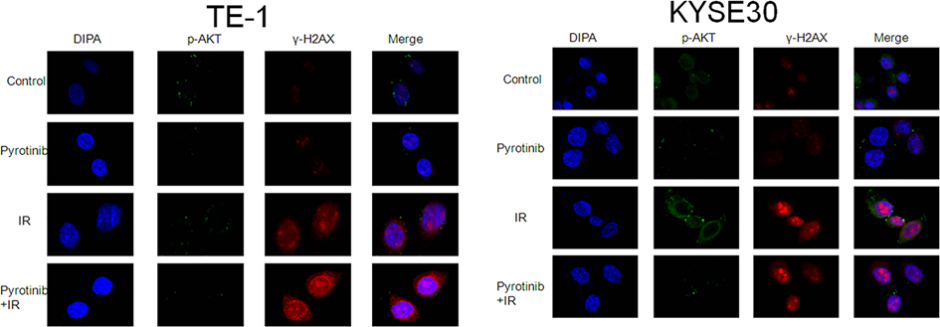
Immunofluorescence detection of pAKT and γ-H2AX expression in esophageal cancer cells Cells were treated with pyrotinib for 24 h or/and 6-Gy irradiation: TE-1 (left) and KYSE30 (right).

Pyrotinib obviously reduced the expression of p-AKT in irradiated and non-irradiated cells. The expression of γ-H2AX was increased after irradiation in these two cell lines, which was not influenced by pyrotinib treatment (Figure 7). Immunofluorescence results showed a co-localization of p-AKT with γ-H2AX after irradiation in both TE-1 and KYSE30 cells, which could also be reduced by pyrotinib treatment (Figure 8).

## Discussion

We examined the effect of pyrotinib combined with radiotherapy on two esophageal cancer cell lines that highly express HER2. HER2 is an EGFR family member that possesses tyrosine kinase activity. Tyrosine residues in the cytoplasmic domain of this receptor can be auto-phosphorylated by dimerization, resulting in activation of various signaling pathways related to cell proliferation and tumorigenesis [4]. Pyrotinib is an irreversible HER2 tyrosine kinase inhibitor that showed anti-tumor effect in breast cancer [8,9]. Clinical studies are underway to examine the anti-tumor effect of pyrotinib in gastric cancer [10] and lung cancer [11]. Here, we found that pyrotinib significantly reduced HER2 phosphorylation, resulting in reduced cell proliferation of TE-1 and KYSE30 cells. This effect was in accordance with a previous report that pyrotinib reduced HER2 phosphorylation in lung cancer patient-derived xenografts and inhibit cell growth of dissociated organoid cells [11]. Our findings indicated that pyrotinib could inhibit cell proliferation of HER2-positiveesophageal cancer cells by inhibiting HER2 phosphorylation.

Radiation resistance in esophageal cancer patients may cause radiation therapy failure and lead to a less promising prognosis [8]. As HER2 was found to be associated with adaptive radiation resistance, while HER2 inhibition was found to reduce radioresistance in breast cancer [6,7]; here, we examined whether pyrotinib could enhance the effect of radiation in esophageal cancer cells. Results showed that compared with radiation treatment alone, cells subjected to pyrotinib combined with radiation treatment showed reduced cell proliferation, indicating a radiosensitization effect of pyrotinib in esophageal cancer cells.

Next, we investigated the possible mechanisms underlying the radiosensitization effect of pyrotinib. It is well documented that cell cycle is related to cellular response after radiation [12]. Previous study found that the duration of G2 phase of the cell cycle was dose-dependently increased by radiation [13]. Consistently with this result, we found that radiation significantly increased the duration of G2/M phase in esophageal cancer cells. Treatment with pyrotinib significantly reduced the radiation-induced G2/M arrest in these two cell lines. As G2 arrest plays a critical role in DNA repair [12] and G2 arrest was considered to play a role in cell survival after radiation [14], pyrotinib might enhance radiation-induced cell damage by reducing G2 duration. DNA double-strand break (DSB) induced by radiation is the main cause of cell death after radiation. Here, we examined the radiation-induced DNA-DSBs by measuring the expression of γ-H2AX. Results showed that γ-H2AX expression was obviously increased after radiation. Although pyrotinib treatment did not further increase γ-H2AX expression, we found that pyrotinib enhanced the radiation-induced decrease in p-Akt expression. Preclinical studies have shown that activation of Akt pathway could accelerate repair of DNA-DSB and improve cell survival after radiation [15]. Our results suggested that pyrotinib enhance the effect of radiation on esophageal cancer cells via reducing DNA repair.

On the other hand, we found that pyrotinib increased the duration of G0/G1 phase. Cyclin D1/CDK4 complexes are crucial for regulating the G1 to S progression in the cell cycle and stimulating cell growth, while reduction of Cyclin D1/CDK4 levels were shown to restrain the G0/G1 to S progression [16]. Previous study showed high expression of cyclins D1 in HER2 overexpressing breast cancer and pyrotinib treatment potently decrease the protein level of Cyclin D1 and CDK4 in HER2 positive human breast cancer cell lines [17]. Consistently, we found that pyrotinib could reduce cyclin D1 and CDK4 levels in esophageal cancer cells. Our results suggested that pyrotinib could enhance the anti-proliferation effect of radiation by increasing G0/G1 arrest, via regulating cell cycle related proteins. However, further studies are required to examine the detailed mechanism and possible pathways underlying this effect. Moreover, besides the proteins tested in the present study, other genes related to HER2 activation signaling pathway and radiation sensitivity should be studied to explore further mechanisms regarding the radiosensitization effect of pyrotinib in HER2-positive esophageal cancer cells. Another meaningful future work would experiment directly measuring the relationship between the activity of the target proteins and cell cycle arrest and DNA damage repair.

## Conclusions

Pyrotinib inhibitd HER2 activation and exerted anti-proliferative effect in human esophageal cancer cells. Furthermore, it sensitivated HER2 positive esophageal cancer cells to radiation treatment, enhancing the anti-proliferative effect of radiation. These effects might be via the inhibition of HER2 phosphorylation, regulation of cell cycle distribution, and affection of the protein expression that is related to DNA repair. These findings are expected to provide new therapeutic strategy for the application of new drugs for esophageal cancer.

## Supplementary Material

Supplementary Figure S1

## Abbreviations

DSB: double-strand break
EGFR: epidermal growth factor receptor
HER2: human epidermal growth factor receptor 2
