# Supplementary material for: Radiosensitization of HER2-positive esophageal cancer cells by pyrotinib
Source: Biosci Rep. 2020 Feb 18;40(2):BSR20194167. doi: 10.1042/BSR20194167 (PMC7029153; doi:10.1042/BSR20194167)
Supplement: Supplementary Figure S1 [file BSR-2019-4167_supp.pdf]

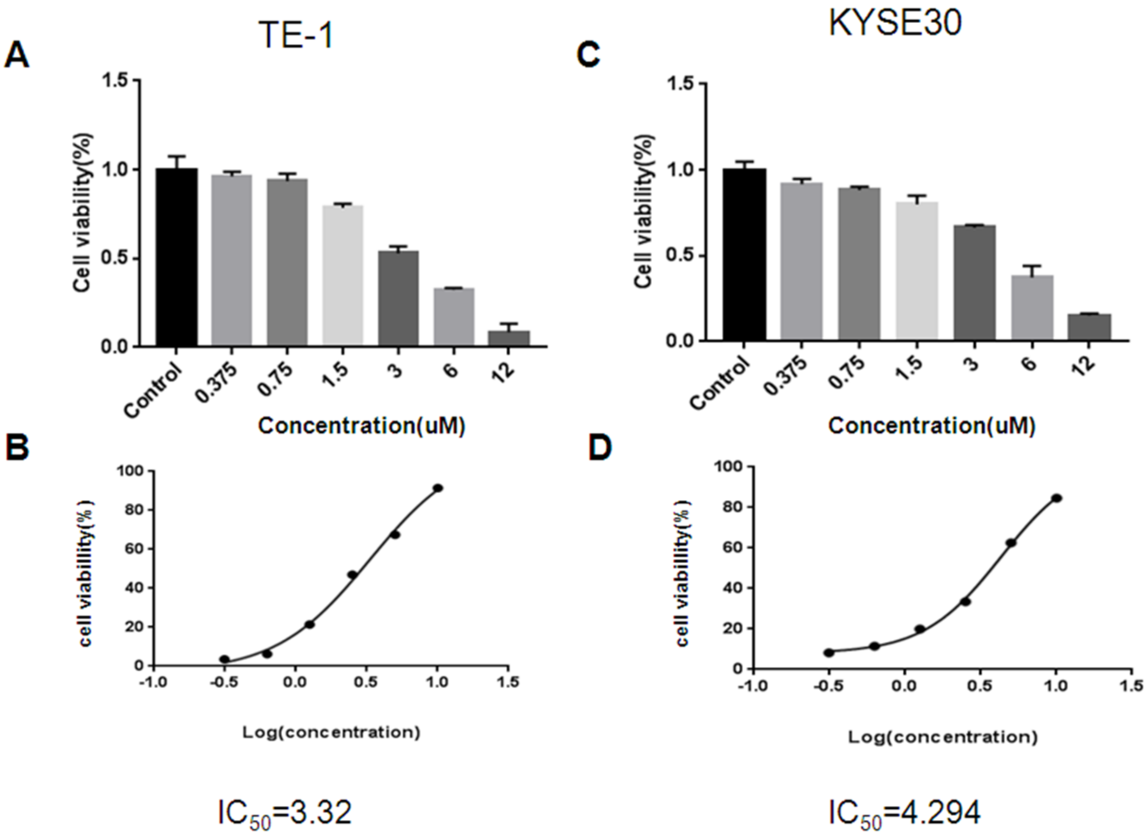

Supplementary Figure 1 Cytotoxic effect of pyrotinib on human esophageal cancer cell lines.

A. TE-1 cell concentration vs. cell viability; B. Logic plot of the TE-1 cell concentration vs. different cell viability; C. KYSE30 cell concentration vs. cell viability; D. Logic plot of the KYSE30 cell concentration vs. different cell viability.
